# Cohesive Energy Densities Versus Internal Pressures of Near and Supercritical Fluids

**DOI:** 10.3390/molecules24050961

**Published:** 2019-03-08

**Authors:** Michal Roth

**Affiliations:** Institute of Analytical Chemistry of the Czech Academy of Sciences, Veveří 97, 60200 Brno, Czech Republic; roth@iach.cz; Tel.: +420-532-290-171

**Keywords:** cohesive energy density, internal pressure, supercritical fluid, tunable solvent, equation of state, water

## Abstract

Over half a century ago, Wiehe and Bagley suggested that a product of the internal pressure and molar volume of a liquid measures the energy of nonspecific intermolecular interactions whereas the cohesive energy reflects the total energy of intermolecular interactions in the liquid. This conjecture, however, has never been considered in connection with near and supercritical fluids. In this contribution, the cohesive energy density, internal pressure and their ratios are calculated from high precision equations of state for eight important fluids including water. To secure conformity to the principle of corresponding states when comparing different fluids, the calculations are carried out along the line defined by equality between the reduced temperature and the reduced pressure of the fluid (*T*_r_ = *P*_r_). The results provide additional illustration of the tunability of the solvent properties of water that stands apart from those of other near and supercritical fluids in common use. In addition, an overview is also presented of the derivatives of cohesive energy density, solubility parameter and internal pressure with respect to temperature, pressure and molar volume.

## 1. Introduction

Among all single-component solvents used in supercritical fluid technology, water has been recognized as the solvent with the greatest degree of tunability of the solvating abilities through changes in operating temperature and pressure [1,2]. Because of unique features of water and its importance as a solvent, a wide range of correlations have been developed for the important properties of water including relative permittivity [3,4] and ion product [5,6]. The variations in relative permittivity and ion product have been widely used to discuss/interpret the variable solvent properties of water. For example, at ambient conditions of 298.15 K and 0.1 MPa, the relative permittivity and ion product of water are 78.4 and 1.01 × 10^−14^ [mol.kg^−1^]^2^, respectively, whereas, at supercritical conditions of 773.15 K and 30 MPa, the relative permittivity and ion product of water drop to mere 1.7 and 1.2 × 10^−21^ [mol.kg^−1^]^2^, respectively [4,5]. These values illustrate that the solvent properties of water can be tuned within wide limits by properly adjusting the operating temperature and pressure. When interpreting and quantifying the solvent polarities in a broader sense, solvent effects on the absorption spectra of suitable organic probe molecules have also been frequented [7].

Another line of thought when considering the solvent properties may be based on strictly thermodynamic (*PvT*) properties of fluids. Over half a century ago, Wiehe and Bagley [8] suggested that a product of the internal pressure and molar volume of a liquid measures the energy of nonspecific intermolecular interactions whereas the cohesive energy reflects the total energy of intermolecular interactions in the liquid. To illustrate the concept of Wiehe and Bagley, let us recall the definition of cohesive energy density *c*,

(1) $$c=\frac{u^{0}-u}{v}$$

where *v* is the fluid’s molar volume, *u* is the fluid’s molar internal energy and *u*^0^ is the molar internal energy of the fluid in an ideal gas state at the particular temperature. The numerator in equation (1), *u*^0^ − *u*, is called cohesive energy and, obviously, it is the energy needed to break all intermolecular interactions in the fluid, whatever their kind. In turn, the internal pressure *P*_int_ of a fluid is the isothermal derivative of the fluid’s internal energy with respect to the fluid’s volume at a constant temperature,

(2) $$P_{\mathrm{int}}={\left(\frac{\partial u}{\partial v}\right)}_{T}=T{\left(\frac{\partial P}{\partial T}\right)}_{V}-P=T\gamma_{V}-P$$

where *T* is the temperature, *P* is the pressure, *V* is the volume and γ*_V_* is the thermal pressure coefficient. Wiehe and Bagley [8] assumed that, upon an infinitesimal change of volume, only nonspecific interactions in the fluid are affected, with specific interactions (hydrogen bonds) remaining intact. Wiehe and Bagley applied their concept to common liquid solvents near ambient temperature and pressure and, to the best of the present author’s knowledge, extension of the concept to near- and supercritical fluids has never been considered although the validity of Equations (1) and (2) is not limited to the liquid state. It follows from the Wiehe and Bagley’s original concept that the ratio *c*/*P*_int_ may serve as an approximate measure of the strength of total intermolecular interactions relative to the strength of nonspecific interactions.

The purpose of the present contribution is to discuss the behavior of *c*, *P*_int_ and *c*/*P*_int_ of eight popular fluids in their near and supercritical states to illustrate the unique position of water from yet another angle of view. To secure conformity to the principle of corresponding states when comparing different fluids, cohesive energy density, internal pressure and their ratio are calculated for the eight fluids along the line defined by equality between the reduced temperature and the reduced pressure of the fluid (*T*_r_ = *P*_r_). An important feature of this choice is that the *T*_r_ = *P*_r_ line does not interfere with the vapor–liquid coexistence curve of the fluid (except at the critical point itself, *T*_r_ = *P*_r_ = 1). In addition, an overview is also presented of the derivatives of cohesive energy density, solubility parameter [9] and internal pressure with respect to temperature, pressure and molar volume.

It should also be noted here that the physical dimension of both internal pressure *P*_int_ and cohesive energy density *c* is pressure; therefore, the ratio *c*/*P*_int_ is dimensionless. In fact, the unit J.cm^-3^, often used for the cohesive energy density, is the same as MPa.

## 2. Results

In this section, the calculated results are discussed in the sequence starting from the thermal pressure coefficient γ*_V_*, and continuing through the internal pressure *P*_int_ and the cohesive energy density *c* to the ratio *c*/*P*_int_.

### 2.1. Thermal Pressure Coefficient

**Figure 1:**
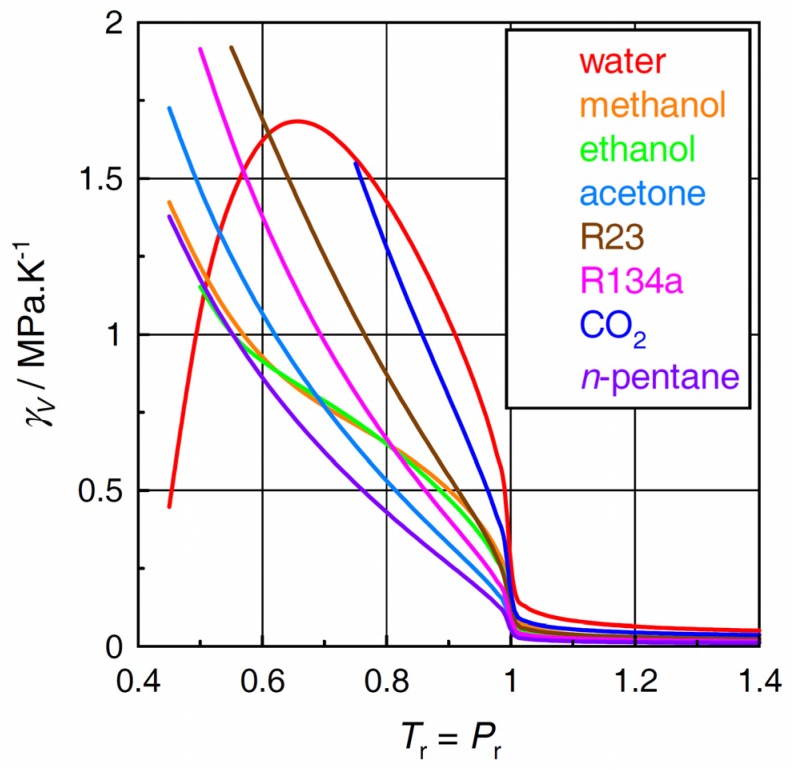
Thermal pressure coefficients γ*_V_* of the selected fluids.

The plot in Figure 1 shows the calculated values of the thermal pressure coefficient as this quantity is an important constituent of the internal pressure of the fluid (see Equation (2)). It is readily apparent that, along the *T*_r_ = *P*_r_ line, the thermal pressure coefficient of water behaves very differently from those of the other fluids. The “hump” in the curve for water is only partly paralleled, to a much smaller extent, in the curves for methanol and ethanol while being absent in the other fluids.

### 2.2. Internal Pressure

**Figure 2:**
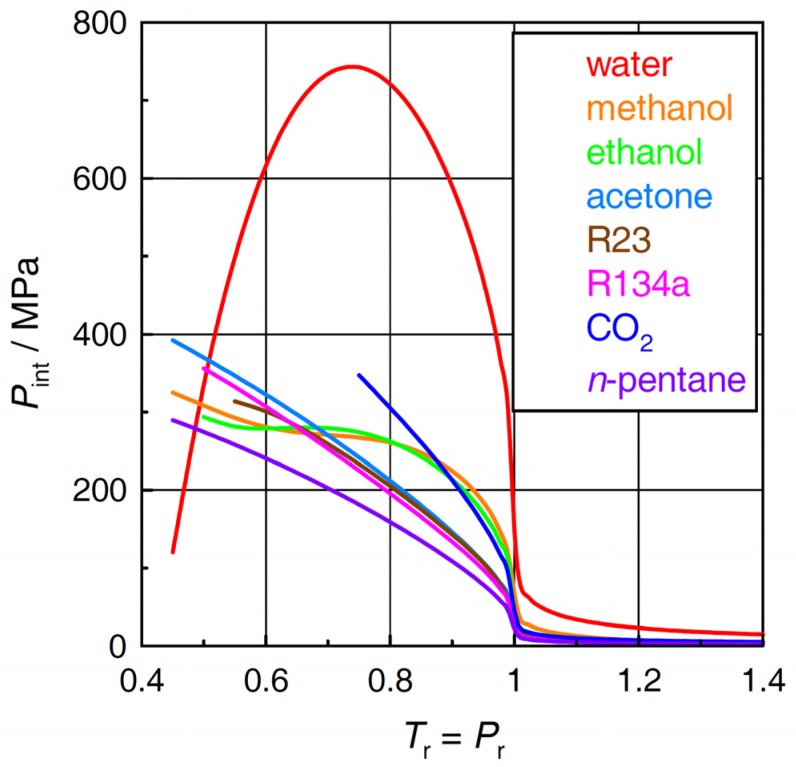
Internal pressures *P*_int_ of the selected fluids.

The patterns in γ*_V_* in the individual fluids (Figure 1) translate themselves into the respective plots for the internal pressure via Equation (2). Figure 2 indicates that the different behavior of the internal pressure between water and the other fluids is even more pronounced than the difference in the thermal pressure coefficient shown in Figure 1. It should be noted that the normal boiling temperature of water (100 °C, 373.15 K) corresponds to *T*_r_ = 0.5767. The maximum in the curve for water in Figure 2 appears at a temperature around 200 °C. Again, the plots for methanol and ethanol also show upward-convex regions although far less significant as compared with water.

### 2.3. Cohesive Energy Density

**Figure 3:**
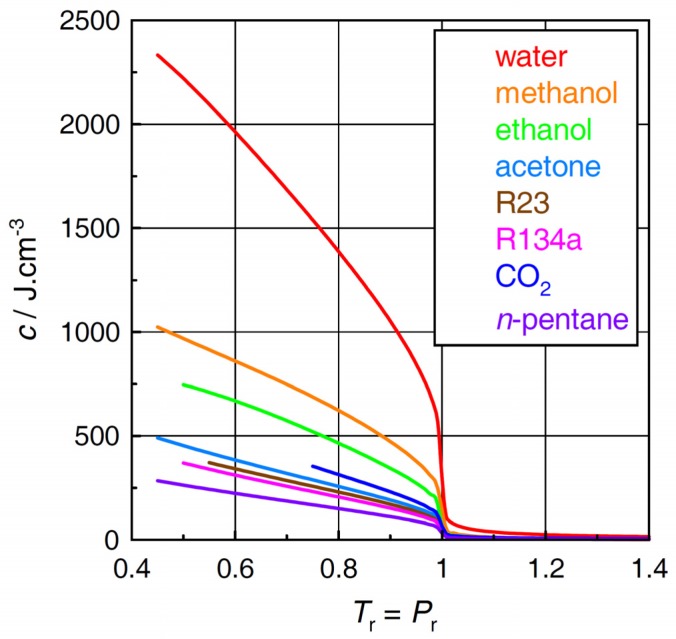
Cohesive energy densities *c* of the selected fluids.

Unlike the plots in Figure 1, Figure 2 and Figure 3 suggests that, in the plots of cohesive energy density, there is no qualitative difference between water and the other fluids. Note, however, that the cohesive energy density of water exceeds those of the other fluids even at slightly supercritical conditions (*T*_r_ > 1).

### 2.4. Ratio of Cohesive Energy Density to Internal Pressure

**Figure 4:**
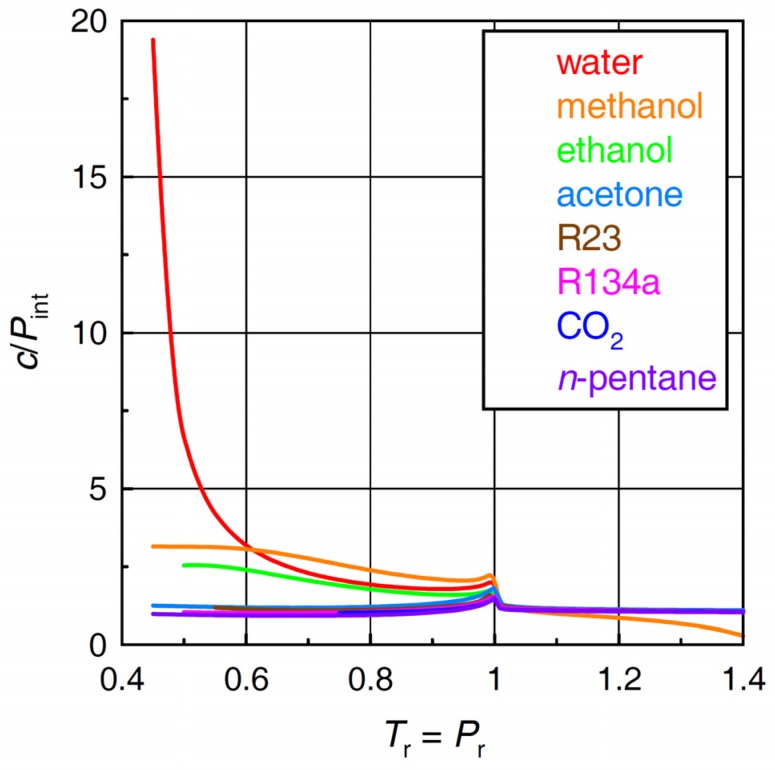
Cohesive energy density/internal pressure ratios *c*/*P*_int_ of the selected fluids.

As shown in Figure 4, the ratio *c*/*P*_int_ of water behaves very differently from those of the other fluids although, in fact, the difference occurs outside the domain of “near- and supercritical fluid”. At low subcritical temperatures (*T*_r_ < 0.6), *c*/*P*_int_ values of water far exceed those of the other fluids. In accord with the original concept of Wiehe and Bagley [8], *c*/*P*_int_ values are also rather high in methanol and ethanol, most probably because of intermolecular hydrogen bonding. Along the *T*_r_ = *P*_r_ line, the drop in *c*/*P*_int_ of water with rising temperature is much steeper as compared with methanol and ethanol, and, at *T*_r_ ~ 0.61, *c*/*P*_int_ of water drops even below that of methanol. This finding may appear somewhat surprising given the fact that the cohesive energy of water (see Figure 3) exceeds those of the other fluids even at supercritical conditions. As the temperature increases above the critical (*T*_r_ > 1), *c*/*P*_int_ converges to unity in most fluids including water. In the frame of Wiehe and Bagley’s concept [8], this finding would suggest that specific intermolecular interactions are no longer important in the near- and supercritical fluid region. The different course seen in methanol is likely due to a technical issue in the equation of state rather than to a fundamental feature of intermolecular interactions in methanol.

## 3. Materials and Methods

Comparative calculations of thermodynamic properties of different pure fluids have often been based on high precision equation-of-state packages such as ThermoFluids [10] or NIST Refprop [11]. Here, the calculations for the eight fluids have been carried out using the ThermoFluids software package [10] with embedded original equations of state or data for acetone [12], carbon dioxide [13], ethanol [14], methanol [15], *n*-pentane [16], R23 (trifluoromethane, fluoroform) [17], R134a (1,1,1,2-tetrafluoroethane) [18] and water [19]. In the calculations, the step in reduced temperature and pressure was 0.05 except between *T*_r_ = *P*_r_ from 0.95 to 1.05 where the step was 0.01. The upper limit of *T*_r_ = *P*_r_ was 1.40 in all fluids. The default value of the lower limit was 0.45, and it was applied in acetone, methanol, *n*-pentane and water. In the other fluids, this lower limit would fall within the solid phase region so that higher values had to be applied: 0.50 in ethanol and R134a, 0.55 in R23 and 0.75 in carbon dioxide, respectively. Calculation of the cohesive energy requires the molar internal energy in an ideal gas state. In this calculation, pressure was set to 1 × 10^−6^ Pa in all fluids at all temperatures. The calculated results have been available in the Supplementary Materials. All plots of the calculated properties have been prepared with the KyPlot graphing software (http://www.kyenslab.com), and the calculated data have been smoothed with piecewise cubic polynomials.

## 4. Discussion

Calculations based on high precision equations of state suggest that, at low subcritical temperatures along the *T*_r_ = *P*_r_ line, the ratio of cohesive energy density to internal pressure in water is significantly higher than in the other solvents. From a purely thermodynamic point of view, the reason for this is embedded in specific behavior of the thermal pressure coefficient of water along the *T*_r_ = *P*_r_ line. Some features similar to but much less important than the properties of water can also be seen in methanol and ethanol. These findings indicate that variations of the solvent properties of methanol and ethanol with temperature and pressure are far less important as compared with water. The calculated values of the cohesive energy density/internal pressure ratio provide yet another illustration of unique position of water among other near- and supercritical fluid solvents.

The weakest point of the present treatment may reside in the modeling of internal pressure; despite the century-long efforts spent on this problem [20,21,22], it still does not appear to have been resolved conclusively as regards the modeling of internal pressure in a wide range of temperature and pressure [23,24].

The variations of cohesive energy density and internal pressure of a fluid with state variables (*PvT*) usually have not received much attention, although they are quite important and an insight into their physical background seems desirable. In the Appendix A, therefore, an overview is presented of the derivatives of cohesive energy density, solubility parameter and internal pressure with respect to temperature, pressure and molar volume of the fluid. The expressions have been derived from Equations (1) and (2) employing standard thermodynamic relationships, and a list of symbols has been compiled at the end of Appendix A. It should also be mentioned here that the quantities appearing in Equations (A1)–(A15) are accessible from high precision equation-of-state packages such as ThermoFluids [10] or NIST Refprop [11], either directly or numerically (such as the derivatives of α*_P_*, β*_T_*, γ*_V_*, *c_P_* and *c_V_*).

## 5. Conclusions

The calculations suggest that the ratio of cohesive energy density and internal pressure of water varies within a much wider range as compared with the other fluids included in this study. However, a large part of *c*/*P*_int_ variations with temperature and pressure occurs at low subcritical temperatures, that is, outside the domain of near- and supercritical fluid. In a broader sense, therefore, applicability of Wiehe and Bagley’s original concept remains confined to subcritical liquids because, in the near- and supercritical region, the ratio of cohesive energy density and internal pressure approaches unity. Within the frame of Wiehe and Bagley’s concept, this finding would suggest that specific intermolecular interactions become unimportant in the near- and supercritical region.

## Supplementary Materials

The following are available online: A MS Excel file containing numerical values of all calculated properties needed to construct the plots in Figures 1–4.

## Appendix A

### Derivatives of c and P_int_ with Respect to T, P and v

The derivatives of cohesive energy density *c* are given by

(A1) $${\left(\frac{\partial c}{\partial T}\right)}_{v}=\frac{c_{V}^{0}-c_{V}}{v}$$

(A2) $${\left(\frac{\partial c}{\partial T}\right)}_{P}=\frac{c_{V}^{0}-c_{V}}{v}-\alpha_{P}\left(P_{\mathrm{int}}+c\right)$$

(A3) $${\left(\frac{\partial c}{\partial P}\right)}_{T}=\beta_{T}\left(P_{\mathrm{int}}+c\right)$$

(A4) $${\left(\frac{\partial c}{\partial P}\right)}_{v}=\frac{c_{V}^{0}-c_{V}}{v\gamma_{V}}$$

(A5) $${\left(\frac{\partial c}{\partial v}\right)}_{T}=-\left(P_{\mathrm{int}}+c\right)/v$$

(A6) $${\left(\frac{\partial c}{\partial v}\right)}_{P}=\frac{1}{v}\left[\frac{c_{V}^{0}-c_{V}}{v\alpha_{P}}-P_{\mathrm{int}}-c\right]$$

The temperature derivative of cohesive energy density along the vapor–liquid coexistence curve may be written as

(A7) $${\left(\frac{\partial c}{\partial T}\right)}_{\sigma }=\frac{c_{V}^{0}-c_{V}}{v}-\left[\beta_{T}{\left(\frac{\partial P}{\partial T}\right)}_{\sigma }-\alpha_{P}\right]\left(P_{\mathrm{int}}+c\right)$$

with (∂*P*/∂*T*)*_σ_* being the slope of the vapor-liquid coexistence curve. With the proper values inserted, Equation (A7) is valid for both liquid and vapor phases.

Since the solubility parameter δ of a fluid has been defined as the square root of the cohesive energy density, the derivatives of the solubility parameter are readily obtained from the corresponding derivatives described by Equations (A1)–(A7). Generally,

(A8) $$\frac{\partial \delta }{\partial x}=\frac{1}{2\delta }\frac{\partial c}{\partial x}$$

where ∂*c*/∂*x* is any of the derivatives given by Equations (A1)–(A7).

The derivatives of the internal pressure may be described as follows,

(A9) $${\left(\frac{\partial P_{\mathrm{int}}}{\partial T}\right)}_{v}=T{\left(\frac{\partial \gamma_{V}}{\partial T}\right)}_{v}={\left(\frac{\partial c_{V}}{\partial v}\right)}_{T}=T{\left(\frac{\partial^{2}P}{\partial T^{2}}\right)}_{v}$$

(A10) $${\left(\frac{\partial P_{\mathrm{int}}}{\partial T}\right)}_{P}=\gamma_{V}+T{\left(\frac{\partial \gamma_{V}}{\partial T}\right)}_{P}=\gamma_{V}+{\left(\frac{\partial c_{P}}{\partial v}\right)}_{T}$$

(A11) $${\left(\frac{\partial P_{\mathrm{int}}}{\partial P}\right)}_{T}=T{\left(\frac{\partial \gamma_{V}}{\partial P}\right)}_{T}-1=-T\left[\alpha_{P}+v{\left(\frac{\partial \alpha_{P}}{\partial v}\right)}_{T}\right]-1$$

(A12) $${\left(\frac{\partial P_{\mathrm{int}}}{\partial P}\right)}_{v}=T{\left(\frac{\partial \gamma_{V}}{\partial P}\right)}_{v}=T\left[\frac{1}{T\gamma_{V}}\left\{{\left(\frac{\partial c_{P}}{\partial v}\right)}_{T}-\frac{c_{P}}{\gamma_{V}}{\left(\frac{\partial \gamma_{V}}{\partial v}\right)}_{T}\right\}-\alpha_{P}-v{\left(\frac{\partial \alpha_{P}}{\partial v}\right)}_{T}\right]$$

(A13) $${\left(\frac{\partial P_{\mathrm{int}}}{\partial v}\right)}_{T}=T{\left(\frac{\partial \gamma_{V}}{\partial v}\right)}_{T}+\frac{1}{v\beta_{T}}=\frac{1}{v\beta_{T}}\left[\frac{T}{\beta_{T}}{\left(\frac{\partial \beta_{T}}{\partial T}\right)}_{v}+1\right]$$

(A14) $${\left(\frac{\partial P_{\mathrm{int}}}{\partial v}\right)}_{P}=\frac{\gamma_{V}}{v\alpha_{P}}+T{\left(\frac{\partial \gamma_{V}}{\partial v}\right)}_{P}=\frac{1}{v\beta_{T}}+T{\left(\frac{\partial \gamma_{V}}{\partial v}\right)}_{P}$$

The temperature derivative of internal pressure along the vapor–liquid coexistence curve is given by

(A15) $${\left(\frac{\partial P_{\mathrm{int}}}{\partial T}\right)}_{\sigma }=\gamma_{V}+T{\left(\frac{\partial \gamma_{V}}{\partial T}\right)}_{P}+\left[T{\left(\frac{\partial \gamma_{V}}{\partial P}\right)}_{T}-1\right]{\left(\frac{\partial P}{\partial T}\right)}_{\sigma }\;=\gamma_{V}+{\left(\frac{\partial c_{P}}{\partial v}\right)}_{T}-\left\{T\left[\alpha_{P}+v{\left(\frac{\partial \alpha_{P}}{\partial v}\right)}_{T}\right]+1\right\}{\left(\frac{\partial P}{\partial T}\right)}_{\sigma }$$

### List of Symbols

*c*	cohesive energy density
*c_P_*	molar isobaric heat capacity
*c_V_*	molar isochoric heat capacity
*c_V_* ^0^	ideal-gas molar isochoric heat capacity
*P*	pressure
*P*_int_	internal pressure [= (∂*u*/∂*v*)*_T_*]
*T*	temperature
*u*	molar internal energy
*u* ^0^	ideal-gas molar internal energy
*V*	volume
*v*	molar volume

### Greek Symbols

*α_P_*	isobaric expansivity [= (1/*v*)(∂*v*/∂*T*)*_P_*]
*β_T_*	isothermal compressibility [= −(1/*v*)(∂*v*/∂*P*)*_T_*]
γ*_V_*	thermal pressure coefficient [= (∂*P*/∂*T*)*_V_*]
*δ*	solubility parameter [= *c*^1/2^]
